# Evaluation of thiol disulfide balance in adolescents with vitamin B12 deficiency

**DOI:** 10.1186/s13052-022-01396-2

**Published:** 2023-01-07

**Authors:** Mehmet Semih Demirtas, Huseyin Erdal

**Affiliations:** 1 Department of Pediatrics, Aksaray University Training and Research Hospital, Aksaray, Turkey; 2 Department of Medical Genetics, Faculty of Medicine, Aksaray University, Aksaray, Turkey

**Keywords:** Vitamin B12, Child, Adolescent, Oxidative stress, Thiol, Homocysteine, Thiol/disulfide balance

## Abstract

**Background:**

Vitamin B12 is an important vitamin for metabolism and affects many mechanisms in the body including neuronal migration, DNA synthesis, neurotransmitter synthesis, brain and cognitive development. Increased oxidative stress in the body leads to the damage of the child development, but also plays a crucial role in the pathogenesis of many diseases encountered in the childhood period. Our aim is to investigate whether or not B12 deficiency is associated with dynamic thiol/disulfide homeostasis in adolescent patients.

**Methods:**

This is a case-controlled observational study consisting of 45 adolescent patients with vitamin b12 deficiency and a control group consisting of 45 healthy adolescent. Patients between 11 and 18 ages who applied to the outpatient clinic for the first time with one of the complaints of headache were selected due to their decreased school performance, dizziness, and fatigue. Hemogram, vitamin B12, homocysteine levels and oxidative stress parameters such as native and total thiol disulfide levels and ratios of disulfide/native thiol, disulfide/total thiol, and native thiol/total thiol were measured from the patients.

**Results:**

Vitamin B12 level was found to be significantly lower in vitamin B12 deficiency group (p < 0.001). The serum disulfide level was found to be 27.5 ± 8.38 in the case group and 20.5 ± 8.36 in the control group (p < 0.001). In the multiple linear regression analysis, it was determined that the independent variables of native thiol, homocysteine and disulfide levels effected of vitamin B12 levels (p < 0.001, p < 0.001, p < 0.005 respectively; R^2^ = 0.62).

**Conclusion:**

The results obtained in terms of the effect of vitamin B12 deficiency on oxidative stress in adolescents are remarkable. The increase in oxidative stress parameters in the patient group may also suggest that oxidative stress plays a vital role in vitamin B12 deficiency in adolescence.

## Background

Vitamin B12 is an essential vitamin that is vital for DNA synthesis, neuronal migration, neurotransmitter synthesis, brain and cognitive development. In case of vitamin B12 deficiency, it may have permanent effects on neuronal (West syndrome, peripheral neuropathy, etc.), hematological and cognitive levels, which may be irreversible due to the deterioration in these developmental processes [1, 2]. Vitamin B12 deficiency in adolescents leads to several disorders including mood and behaviour changes, neurological and hematological disorders and decrease in cognitive functions [1]. Vitamin B12 has two important functions in biochemical/metabolic reactions: First, it is a cofactor of methyl melonyl-CoA mutase enzyme that produces succinyl-CoA from methyl melonyl-CoA by deoxidation, and second, it acts as a cofactor of the methyl transferase enzyme which provides production of methionine from homocysteine (HCY) by remethylation in homocysteine metabolism [3]. As a result of the deoxidation of the mitochondria due to the B12 deficiency, serum methylmalonic acid (MMA) increases. This increase results in the inhibition of fatty acid breakdown and accumulation of fatty acids in the cytosol [5]. The synthesis of methionine, which is one of the important amino acids in protein synthesis, is interrupted by negatively and affecting the remethylating metabolism in the increase of cytoplasm and homocysteine levels. In addition, the production of S-adenosyl methionine, which is formed during the methionine cycle in DNA and RNA synthesis, also decreases during this process [5]. B12 deficiency leads to decrease in glutathione synthesis an important element of antioxidant capacity as well as decrease in ATP production, amino acid and DNA synthesis in mitochondria, and the production of Reactive Oxygen Species [6].

In biological systems, free radicals and antioxidants are in balance called oxidative balance. Oxidative balance is impaired due to the increase in reactive oxygen species in the cells or decrease in antioxidant levels due to pathological processes. This condition is known to be oxidative stress. Studies have shown that oxidative stress and thiol disulfide hemostasis is responsible for the pathogenesis of many diseases [7, 8].

Thiols containing sulfhydryl groups [9] are involved in the prevention of oxidative stress that develops with the deterioration of oxidative balance in cells. When the body encounters oxidation, thiol groups form disulfide structures and then, can be reduced back to thiol groups with the reduction of oxidative stress over time. Body creates a dynamic thiol disulfide balance with this mechanism. Maintaining this balance has a significant role in detoxification systems, signal transmission, mechanism of antioxidant protection and regulation of hormone and enzymatic activities [10]. Recent studies are carried out in childhood diseases regarding the thiol-disulfide balance, which is one of the new markers of the oxidative system and can provide crucial information to physicians in the early diagnosis of childhood diseases and neurodevelopmental regression conditions [11, 12].

To the best of our knowledge, this is the first report for the evaluation of thiol disulfide balance in adolescent patients with vitamin B12 deficiency.

## Methods

### Study design

The study was planned as a case-control study in a single Training and Research Hospital center with a Pediatrics Clinic in order to examine the relationship between vitamin B12 deficiency and thiol/disulfide hemostasis in adolescence patients.

## Study Population

The study was planned by selecting 45 of 141 adolescent patients who applied to Aksaray University Training and Research Hospital Pediatrics Outpatient Clinic. The control group consisted of 45 healthy adolescents without any disease who applied to obtain a sports license.

Patients between the ages of 11–18 were selected among the patients who applied to the outpatient clinic for the first time with one of the complaints of headache, decreased school performance, dizziness, and fatigue. Except for vitamin B12 deficiency, patients without iron deficiency anemia and vitamin D deficiency, without any comorbid disease, and with normal cranial magnetic resonance imaging were selected.

96 patients were not included in the study because 43 patients had upper respiratory tract infections such as sinusitis and flu, 33 patients had vitamin D deficiency and iron anemia, 14 patients had comorbid disease and 2 patients use antidepressant medication. Four patients were also excluded as they were diagnosed with migraine.

## Data Collection

Hemogram, vitamin B12, native and total thiol, disulfide and homocysteine levels of the patients were measured. Serum samples were collected into vacutainer tubes containing EDTA, during routine blood sample taking for the measurement of vitamin B12 and other biochemical parameters and were centrifuged at 1500 g × 10 min after sampling. Then, serum samples were portioned and stored at -80 °C in the Eppendorf tube until the time of assay.

## Measurement of Thiol/ disulfide homeostasis

In this study, native and total thiol levels were measured with a new spectrophotometric method developed by Erel and Neselioglu [10]. Disulfide levels were calculated by the half of the difference between the total and native thiols.

## Measurement of homocysteine levels

Homocysteine levels (6–14 µmol/L) were studied with the colorimetric method (automatic analyser MINDRAY-BS400® device). First, oxidized homocysteine is reduced to free HCY. Free HCY then reacts with a co-substrate catalysed by cycling enzymes and was significantly amplified. Then, NADH converts to NAD + through dehydrogenation. The concentration of HCY in the sample is indirectly proportional to the amount of NADH converted to NAD+.

### Statistical analysis

In this study, the data were analysed by using the SPSS 22.0 (IBM, USA) statistical package program. Shapiro-Wilk test was used to determine the distribution patterns of the variables. Numerical values were expressed as mean ± standard deviation, and categorical values were expressed as n [13]. The mean differences between two independent groups were compared using the student’s t-test; The Mann-Whitney U test was used to compare the median values that did not fit the parametric distribution. The Kruskal-Wallis test was used to evaluate oxidative stress in the age category divided into three groups. One-way ANOVA was used to evaluate differences between the groups due to the variables showed normal distribution. Linear regression analysis was applied to evaluate the relationship between dependent and independent variables. P < 0.05 were considered as statistically significant.

## Ethics

Written informed consent was obtained from all parents after the procedures regarding the study were fully explained to the families. This study was approved by ethical committee of Hatay Mustafa Kemal University (protocol number: 2022/10).

## Results

The study consisted of 90 adolescences, 45 of whom were in the patient group and 45 of them were in the control group. While the mean age was 14.1 ± 1.71 years in the vitamin B12 deficiency group, it was 14.1 ± 1.62 years in the control group and there was no difference in age between the two groups (*p* = 0.21) (Table 1). In the vitamin B12 deficiency group, 21 (46.7%) of the patients were male and 24 (53.3%) of them were female; in the control group, 22 (48.9%) of the participants were male and 23 (51.5%) of them were female, and there was no significant difference in gender distribution (p = 0.83) as in the age factor. In addition, there was no significant difference in hemogram parameters including hemoglobine (Hb), red cell distribution width (RDW) and mean corpuscular volume (MCV) between the two groups (Table 2).

**Table 1:** Demographic features of all participants

Parameter		Patient (n = 45) n %	Control (n = 45) n %	p
**Sex**	**Male**	21 (46.7%)	22 (48.9%)	0.83 ^a^
	**Female**	24 (53.3%)	23 (51.5%)	
**Age**		14.1 ± 1.71	14.1 ± 1.62	0.21^¥^

^a^: Chi-Square testi, ^¥^: Student t test

When the laboratory parameters of the study groups were compared, the serum vitamin B12 level was found to be significantly lower in the vitamin B12 deficiency group (p < 0.001). The serum disulphide level was found to be 27.5 ± 8.38 in the patient group and 20.5 ± 8.36 in the control group (p < 0.001). It was found that HCY ​​and native thiol levels were significantly increased in the B12 deficient group (p < 0.001, p < 0.001, respectively). Total thiol and ratios of disulfide/native, disulfide/total and native /total thiol levels were found to be statistically significant between the patient and control groups (Table 2).

**Table 2:** The distribution of serum vitamin B12, Homocysteine, hemoglobin parametres and Thiol levels of all subjects

Parameter	Patient (n = 45) (Min-Max)	Control (n = 45) (Min-Max)	p
Vitamin B12 (pg/ml)	161 (79–280)	314 (107–451)	**< 0.001**
Homocysteine (µmol/L)	39.3 (22.9–94.9)	17.2 (11.6–37.4)	**< 0.001**
Total Thiol (µmol/L)	457.6 (275–685)	594 (243–718)	**< 0.001**
Native Thiol (µmol/L)	402.6 (243–608)	553 (216–688)	**< 0.001**
Disulfide	27.5 (14–55)	20.5 (12–59)	**< 0.001**
Disulfide/Native Thiol	7.1 (3.6–16.2)	3.9 (1.8–12.6)	**< 0.001**
Disulfide/Total Thiol	6.1 (3.4–12.2)	3.6 (1.7–10.1)	**< 0.001**
Native Thiol/Total Thiol	87.7 (75.6–93.2)	92.9 (79.9–96.6)	**< 0.001**
Hemoglobin	14.1 (11-16.3)	14.2 (12.1–16.5)	0.46
MCV	84 (78–95)	83.5 (77-86.3)	0.13
RDW	13.3 (11.4–15.9)	12.9 (11.8–15.6)	0.62

Vitamin B12 levels were measured 164.4 ± 41.3 and 158 ± 3.2 in males and females by gender in patient group, respectively. When B12 levels of both groups were examined, disulfide was found to be 25.2 ± 7.7 in the male and 29.5 ± 8.5 in the female (p = 0.084) (Table 3). In order to evaluate B12 deficiency in terms of age, the adolescents in the patient group were divided into 3 groups aged 11–13, 14–15 and 16–17. There was no significant difference between the values ​​of the patients in terms of age (Table 3).

**Table 3:** Distribution of vitamin B12, Homocysteine and Thiol parameters in the patient group according to gender and age

Parameter	Male (n = 21)	Female (n = 24)	P_1_^β^	Age1*(n = 19)	Age2**(n = 12)	Age 3***(n = 14)	P_2_^§^
Vitamin B12 (pg/ml)	164.4 (94–211)	178 (79–280)	0.37	161.6 (94–256)	168 (106–224)	155 (79–280)	0.60
Homocysteine (µmol/L)	35.8 (22.9–94.9)	42.4 (23.5–86.5)	0.09	38.6 (22.9–94.9)	34 (23.4–57.1)	44.8 (26.3–86.5)	0.14
Total Thiol (µmol/L)	234.2 (275–594)	478 (325–685)	0.15	461.8 (298–685)	462.4 (275–630)	447.8 (305–658)	0.88
Native Thiol (µmol/L)	383.8 (243–510)	419.1 (266–608)	0.19	405 (243–594)	406 (245–578)	396.2 (266–608)	0.89
Disulfide	25.2 (14–44)	29.5 (15–55)	0.08	28.3 (20-45.5)	28.2 (14–55)	25.7 (15–43)	0.52
Disulfide/Native Thiol	6.8 (3.6–14.6)	7.4 (4.1–16.2)	0.39	7.4 (4.1–14.6)	6.9 (36-11.5)	6.9 (4.1–16.2)	0.69
Disulfide/Total Thiol	5.9 (3.4–11.3)	6.3 (3.8–12.2)	0.39	6.4 (3.8–11.3)	6.1 (3.4–9.3)	5.9 (3.8–12.2)	0.68
Native Thiol/Total Thiol	88.1 (77.4–93.2)	87.3 (75.6–92.4)	0.37	87.2 (77.4–92.4)	87.8 (81.4–93.2)	88.2 (75.6–92.4)	0.68

^**β**^**=** Mann-Whitney U test, ^**§**^**=**Kruskal Wallis test

*Age 1 = The group of patients whose age is between 11–13 years; ** Age 2 = 14–15 years;***Age 3 = 16–17 years

In multivariate statistical analyses, gender, age, total thiol, native thiol, disulfide, hemoglobin, MCV, RDW, and homocysteine associated with vitamin B12 deficiency were included in the linear regression model as candidate risk factors. In the multiple linear regression analysis, it was determined that the independent variables of native thiol, homocysteine and disulfide had effects on vitamin B12 levels (p < 0.001, p < 0.001, p < 0.005 respectively; R^2^ = 0.62).

## Discussion

To the best of our knowledge, this is the first study investigating thiol disulfide balance in adolescent patients with vitamin B12 deficiency. It was found that the native thiol levels were lower in the group with vitamin B12 deficiency, while the disulfide level was significantly higher (p < 0.001, p < 0.001, respectively). HCY level in patient group with vitamin B12 deficiency was determined to be higher (p < 0.001). Moreover, it was determined that native thiol, homocysteine and disulfide parameters were statistically significantly correlated with vitamin B12 deficient adolescents. (p < 0.001, p < 0.001, p < 0.005 respectively; R^2^ = 0.62).

Increasing the use of cell phones and technological devices with high SAR values in adolescents, fast-food consumption, smoking cigarettes and other tobacco products result in the increase in the oxidative stress levels of this age groups [14, 15]. In a study investigating oxidative stress levels in healthy children, it was shown that the levels of prooxidant derivatives of reactive oxygen metabolites (d-ROMs) decreased with age [16]. However, in the results of this study, it was found that the age factor did not have any effect on oxidative stress parameters. (Table 3).

B12 deficiency causes late hematological findings such as macrocytic anemia as a result of ineffective DNA synthesis and impaired erythropoiesis [17]. In the absence of typical macrocytic anemia findings, it is difficult to diagnose vitamin B12 deficiencies in children and adolescents. However, it was reported in a study that central nervous system findings can be seen prominently in children with vitamin B12 deficiency who have normal hematological findings [18]. In this study, no significant difference was found between the groups in terms of Hb, MCV, RDW parameters (p = 0.46, p = 0.13, p = 0.62, respectively) (Table 2).

Vitamin B12 deficiency remains the most common reason of childhood megaloblastic anemia. Early diagnosis and treatment of vit-B12 deficiency in infants and adolescents is important for preventing severe anemia, permanent neurological deficits, and loss of cognitive functions [6, 19]. Cobalamin deficiency reduces the activity of methionine synthase involved in cellular methylation reactions, gene expression and protein synthesis, and increases the oxidative stress of metabolism [9, 20, 21]. Güney et al. [22] studied vitamin B12 deficiency in adult patients, and they showed that oxidative stress did not increase in the vitamin B12 deficient group. In a study on oxidative stress in children aged 4–9 years with cobalamin and iron deficiency anemia, it was shown that thiobarbituric acid derivatives (TBARS), an important marker of oxidative stress, increased [23]. One of the most important results of our study is that native thiol levels were decreased in adolescent patients (p < 0.001). We conclude that the increased oxidative stress in the metabolism due to the decreasing vitamin B12 levels leads to decrease in native thiol levels.

The effects of vitamin B12 deficiency on the central and peripheral nervous systems are explained by the accumulation of homocysteine in the tissue and plasma as a result of the defect in enzymatic reactions [21, 24]. The interruption of methionine synthase activity causes increasing serum homocysteine levels. Increasing HCY not only increases N-methyl-D-aspartate (NMDA) levels and ROS production, but also changes the oxidative stress balance by disrupting hemostasis and causes deterioration in endothelial-derived nitric oxide-mediated vasodilation [24, 25]. Rzepke et al. [26] demonstrated that human epidermal melanocytes treated with a B12 antagonist showed increasing ROS production for over 100%. This situation causes an increase in reactive oxygen species [6], especially by interrupting methionine synthase activity and increasing hydrogen peroxide (H_2_O_2_) production by auto-oxidation of increased homocysteine levels [25]. We also found that homocysteine and disulfide levels affected by vitamin B12 level are significant factors indicating increased oxidative stress (p < 0.001, p < 0.005, respectively; R^2^ = 0.62). We hypothesized that it is important to evaluate the relationship between increasing HCY and oxidative stress in adolescent patients and cognitive function and neurological findings.

## Strengths and limitations

Although our most obvious limitation in this study was the relatively small sample size, the exclusion of concomitant diseases and other anemia conditions in vitamin B12 deficiency samples were also increased the reliability of our study. We think that it will be a precursor to study on the relationship between vitamin B12 deficiency and oxidative stress in childhood and prospective cohorts.

## Conclusion

In conclusion, the increase in HCY and disulfide levels and the decrease in native thiol levels in adolescents with vitamin B12 deficiency indicate that the increase in oxidative stress. Measurement of dynamic thiol disulfide levels will provide important perspectives to the clinician who may encounter some of diseases related to oxidative stress levels.

## Data Availability

The data gathered in this study can be requested from the corresponding author reasonably.

## List of abbreviations

SAM: S-adenosyl methionine
H2O2: Hydrogen peroxide
MMA: Serum methylmalonic acid
TBARS: Thiobarbituric acid derivatives
ROS: Reactive Oxygen Species
HCY: Homocysteine
Hb: Hemoglobine.
NMDA: N-methyl-D-aspartate
d-ROMs: Derivatives of reactive oxygen metabolites
RDW: Red cell distribution width
MCV: Mean corpuscular volume
